# Unanswerable Questions About Images and Texts

**DOI:** 10.3389/frai.2020.00051

**Published:** 2020-07-29

**Authors:** Ernest Davis

**Affiliations:** Department of Computer Science, New York University, New York, NY, United States

**Keywords:** unanswerable questions, visual question answering, question answering, VQA, QA, SQUAD, VizWiz

## Abstract

Questions about a text or an image that cannot be answered raise distinctive issues for an AI. This note discusses the problem of unanswerable questions in VQA (visual question answering), in QA (textual question answering), and in AI generally.

The paper under discussion (Kafle et al., 2019) gives an extensive, detailed, and thoughtful review of the many issues that have to be faced in designing datasets to train and evaluate vision and language (V&L) systems, and avoid “Clever Hans” effects (Henizerling, 2019). It further proposes a number of strategies to address the issues and mitigate the problems.

Some of these problems are not specific to V&L systems, but are instances of problems that are common throughout machine learning (ML)-based AI systems. In particular, Kafle, Shrestha, and Kanan write:

Human beings can provide explanations, point to evidence, and convey confidence in their predictions. They also have the ability to say “I do not know” when the information provided is insufficient. However, almost none of the existing V&L algorithms are equipped with these abilities, making their models highly uninterpretable and unreliable.

In VQA [visual question answering], algorithms provide high-confidence answers even when the question is non-sensical for a given image, e.g., “What color is the horse?” for an image that does not contain a horse can yield “brown” with a very high confidence.

There has been a flurry of recent papers addressing the issue of unanswerable questions, both in visual question answering (VQA) and in text-based question answering (QA). As far as can be judged from citations, these two directions of research have been largely independent; that is, the VQA papers rarely cite QA research and vice versa. This note discusses the problem of unanswerable questions in VQA (section 1) and in QA (section 2) and compares them (section 3); they have some commonalities and many important differences. Section 4 briefly discusses some related work from knowledge-based AI. Section 5 discusses the problem of unsolvable problems and unanswerable questions in AI generally. Section 6 discusses why it is important for AI systems to deal with unanswerable questions.

## 1. Unanswerable Questions in Images

In the last few years many VQA datasets have been created in the computer vision research community (see Kafle et al., 2019 for a review). For the most part, these datasets contain high-quality images that have been posted to web sites, paired with questions that have been constructed, either automatically, or by crowd workers, or by in-house participants (see Gurari et al., 2018, Table 1). In most of these datasets, all of the questions are relevant to the image and answerable from the image. As a result, as stated in the above quote from Kafle, Shrestha, and Kanan, a system that has been trained on such a dataset will *always* output an answer, even if the question is unanswerable, because in every example in its training set, the proper response was to output an answer. Researchers interested in detecting irrelevant and unanswerable questions have therefore had to add these deliberately to their datasets.

**Table 1:** Categorization of unanswerable questions in SQuAD 2.0. From Rajpurkar et al. (2018b).

**Reasoning**	**Description**	**Example**	**%**
		Sentence: “*Several hospital pharmacies have decided to*	
T.1. Negation	Negation word inserted	*outsource high risk preparations …”*	9%
	or removed.	Question: “*What types of pharmacy functions have **never***	
		*been outsourced?”*	
		S: “*the extinction of the dinosaurs …allows the*	
T.2. Antonym	Antonym used	*tropical rainforest to spread out across the continent*	20%
		Q: “*The extinction of what lead to the **decline** of*	
		*rainforests?”*	
	Entity, number, or date	S: “*These values are much greater than the 9-88 cm*	
T.3. Entity Swap	replaced with other	*as projected …in its Third Assessment Report*.	21%
	entity, number, or date.	Q: “*What was the projection of sea level increases in the*	
		***fourth assessment report**?”*	
	Word or phrase is	S: *“BSkyB …waived the charge for subscribers whose*	
T.4. Mutual	mutually exclusive	*package included two or more premium channels.”*	16%
Exclusion	with something for which	Q: “*Which service did BSkyB **give away for free***	
	an answer is present	***unconditionally**?”*	
		S: “*Union forces left Jacksonville and confronted*	
	Asks for condition that	*a Confederate Army at the Battle of Olustee*.	
T.5. Impossible	is not satisfied by	*Union forces then retreated to Jacksonville*	4%.
Condition	anything in the paragraph	*and held the city for the remainder of the war.”*	
		Q: “*After what battle did Union forces leave*	
		*Jacksonville **for good**?”*	
T.6. Other	Other cases where the	S: “*Schuenemann et al. concluded in 2011 that the*	
Neutral	paragraph does not imply	*Black Death …was caused by a variant of Y. pestis.”*	24%
	any answer.	Q: “*Who discovered Y. pestis?”*	
T.7. Answerable	Question is answerable		7%
	(i.e., dataset noise)		

Ray et al. (2016) constructed irrelevant questions by randomly pairing an image from the VQA dataset^1^ (Agrawal et al., 2015) with a question from the same dataset; this technique, of course, mostly generates questions that are entirely unrelated to the associated image.

**Figure 1:**
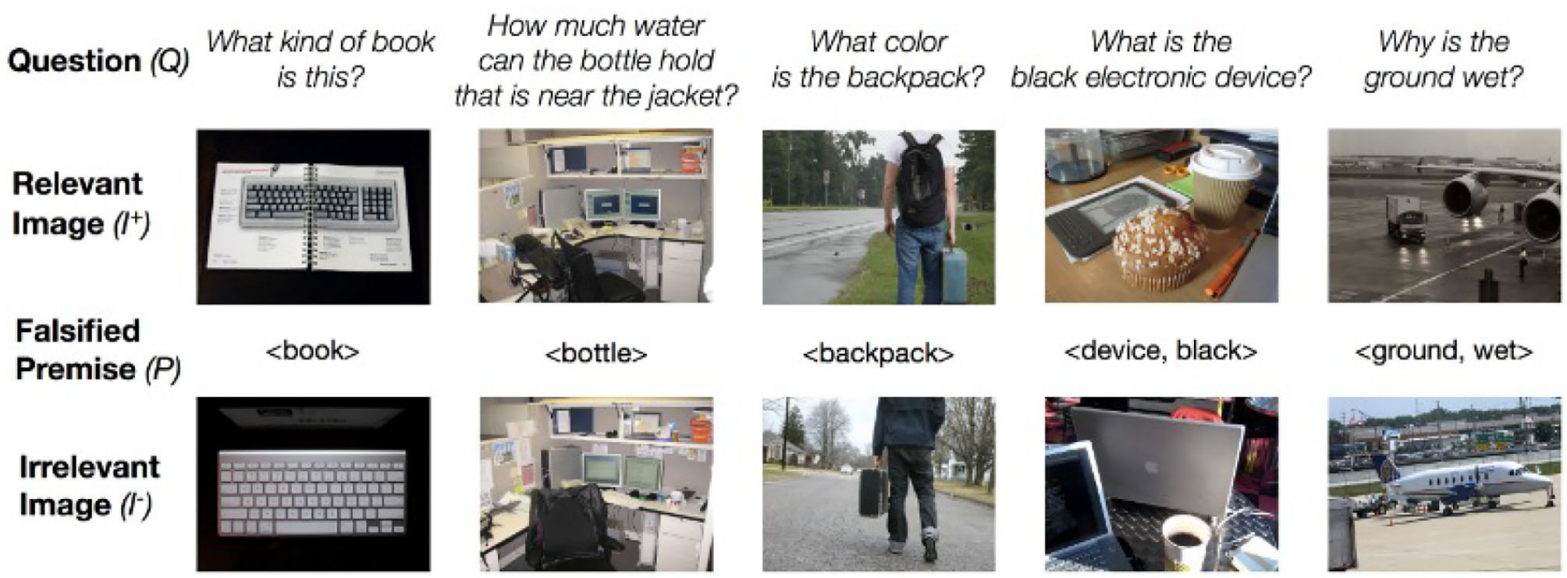
Answerable and Unanswerable Questions in Mahendru et al. (2017). The images are part of the VQA data set http://visualqa.org/ where they are published under a Creative Commons Attribution 4.0 International License.

Mahendru et al. (2017) use a more sophisticated technique. They take an image/question pair 〈*I*^+^, *Q*〉 from the VQA dataset. They then use simple NLP techniques to extract a premise *P* from question *Q*; for instance, from the question “Why is the ground wet?” they extract the premise 〈ground, wet〉. They then search for an image *I*^−^ in the dataset that (a) is visually similar to *I*^+^; (b) violates one aspect of the premise; e.g., an image showing dry ground. The two pairs 〈*I*^+^, *Q*〉 and 〈*I*^−^, *Q*〉 are then added to the database; the discrimination that the first is answerable and the second is unanswerable is generally quite challenging for VQA systems (Figure 1). Mahendru et al. also use the unsatisfied premise to train a system that can give responses that deny the premise e.g., “The ground is not wet.”

**Figure 2:**
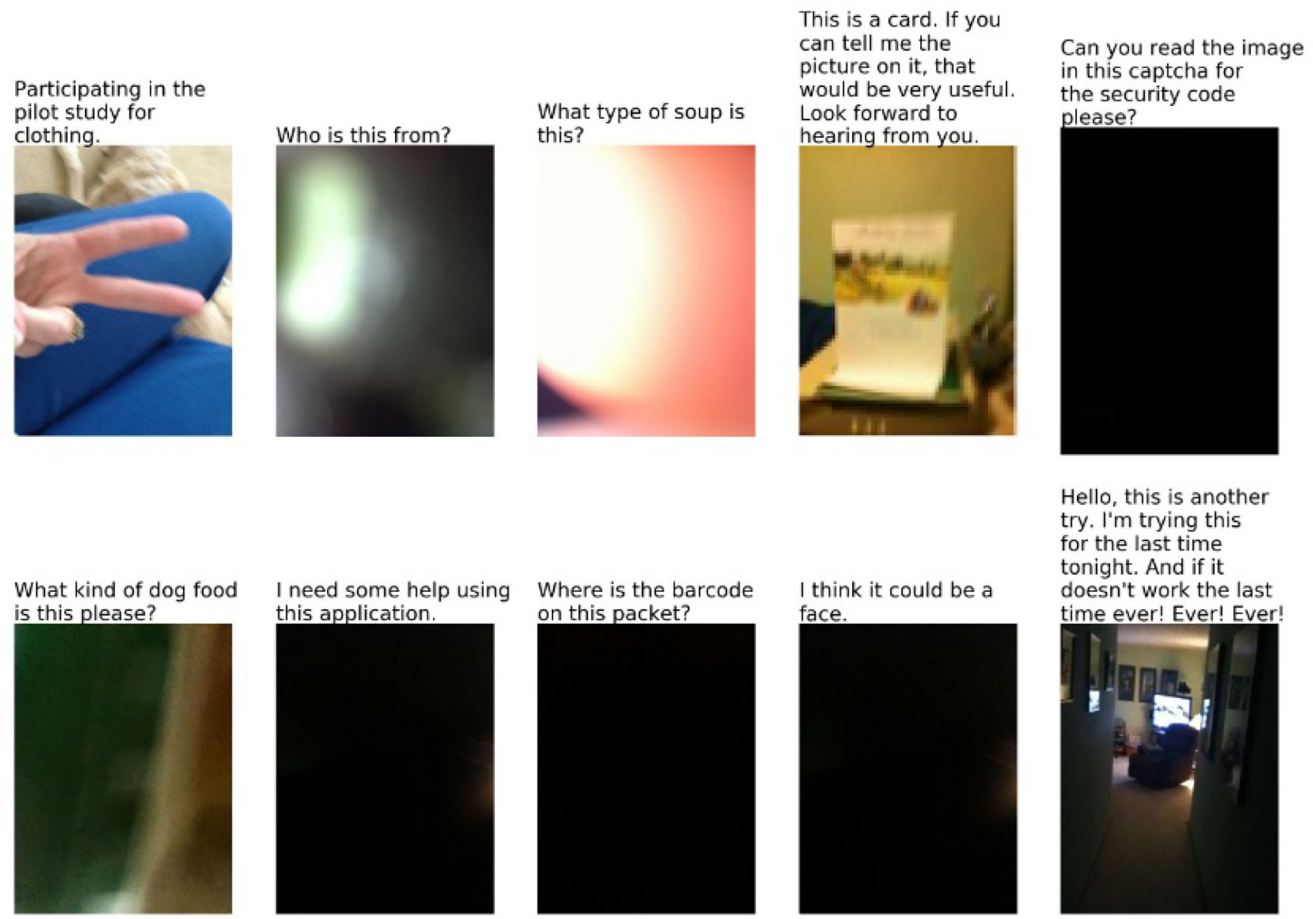
Unanswerable questions in VizWiz. From Gurari et al. (2018). The images and texts are part of the VizWiz data set https://vizwiz.org/tasks-and-datasets/vqa/ where they are published under a Creative Commons Attribution 4.0 International License.

The VizWiz dataset (Gurari et al., 2018) was constructed in an entirely different way from other VQA datasets. VizWiz is an app that allows blind or visually impaired users to take a picture and ask a question about it, which is then answered by human employees or volunteers. With the permission of the users, Gurari et al. collected 31,173 image/questions pairs, after carefully screening by experts to eliminate images that might have personal or embarrassing information. Crowdworkers were then used to write new answers to the questions, or to mark the questions as unanswerable (Figure 2).

The VizWiz dataset has markedly different characteristics from datasets such as VQA:

- The images and questions reflect the practical needs of visually impaired users. This, obviously, yields a very different distribution of images and questions than you get when you download images from the web and then ask people to pose interesting questions about them.
- A significant fraction of the images are of very low quality, due to the users' inability to judge.
- Since the questions are from recorded speech, they are more informal and conversational than the questions in VQA. A significant fraction are missing the first word or two, because the recording started late. Some of them are not questions at all, but other kinds of expressions.

As a result, 28% of the questions in VizWiz are unanswerable.

Other studies that have addressed the issues of unanswerable visual questions include Toor et al. (2017) and Bhattacharya et al. (2019).

**Figure 3:**
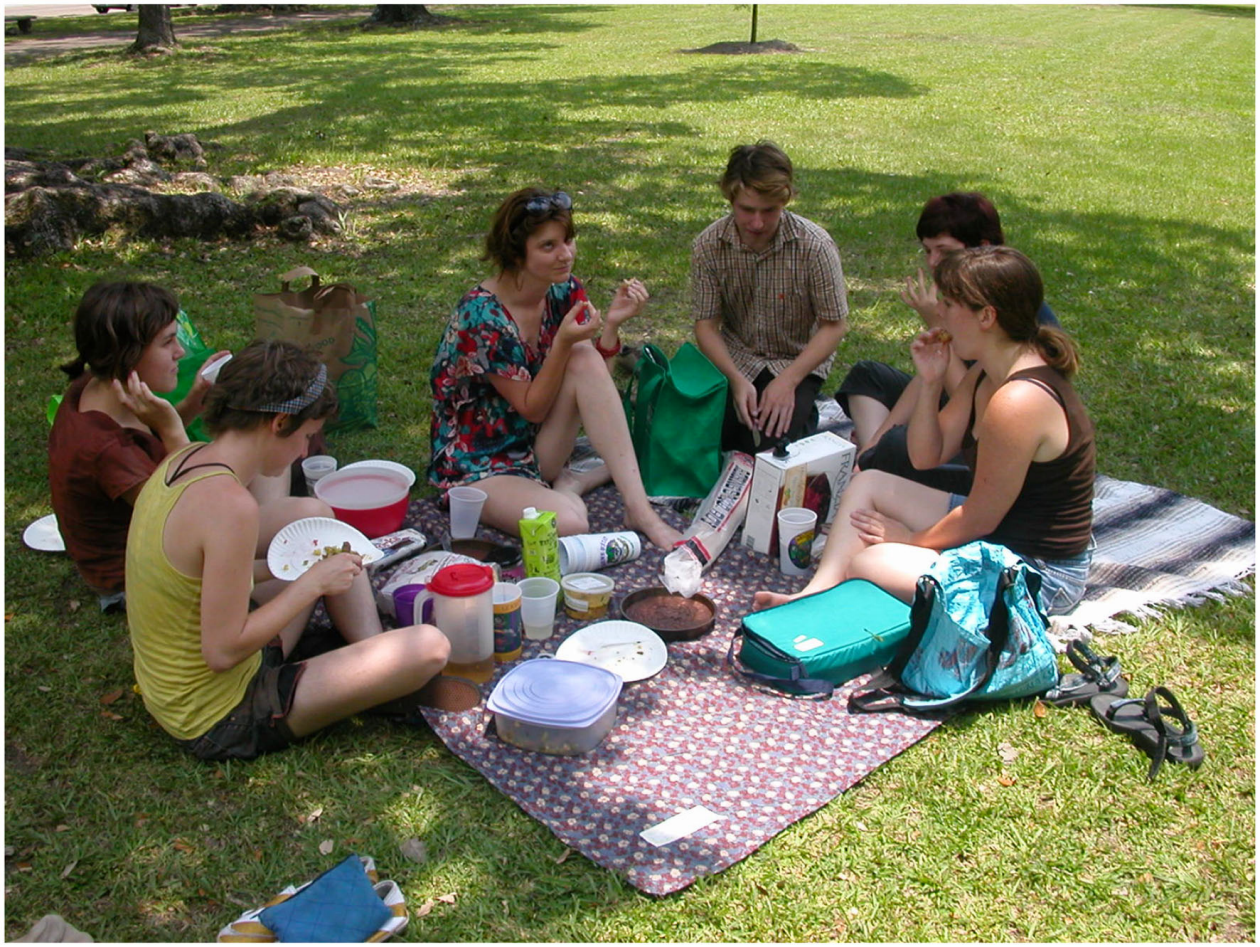
Picnic. From Wikimedia Commons, with a Creative Commons License https://commons.wikimedia.org/wiki/File:Our_pre-July_4th_picnic_NOLA.jpg.

I have not found any dataset that collects visual questions asked by sighted users in a comparably natural setting. Presumably, almost all of these would be questions that the asker cannot themselves easily answer just looking at the image; these are thus “unanswerable questions” at least for this user in this circumstance (there are some exceptions; it is common for someone who is teaching to ask a question for which they know the answer). For instance, looking at the picture of the picnic in Figure 3, one might naturally ask: Who are these people? How do they know each other? What did they have for lunch? Where and when did this take place? What were they talking about? Did they have a good time?

### 1.1. Categories of Unanswerable Questions About Images

Unanswerable questions in VQA can be categorized in terms of the reason that they are unanswerable (these categories derive in part from the discussions in the papers cited above and are in part original. In particular, Bhattacharya et al. (2019) has a comparable list of categories of questions for which crowdworkers have supplied a number of different answers). Where not otherwise specified, the questions below refer to Figure 3.

**V.1. Details that are hard or impossible to discern:** *Is the woman in the center wearing lipstick? What colors are the eyes of the man in the center? What is the food on the plate of the woman with the yellow-green shirt?*
**V.2. Information that is occluded:** *Is the rightmost woman in the brown shirt barefoot? Is there lemonade in the plastic pitcher with the red lid?*
**V.3. Information that is out of the picture frame:** *What kind of tree is the trunk visible in the top left corner? What is casting the shadow on the front edge of the blanket?*
**V.4. Indeterminate spatial relations:** *Is it more than 20 feet from the tree trunk in the top left to the vertical pole in the top center? Is the cardboard box touching the plastic cup that is in front of it? Who is taller: The woman in the center or the woman on the far right?*
**V.5. Information that is (probably) indicated in the photo, to a viewer with the appropriate general knowledge:** *What is the material of the white rug with the black stripes? In what decade was the photo taken?*
**V.6. Information of a kind that is indicated in some photos but doesn't happen to be in this photo:** *What are the people eating for lunch? Who is the youngest person at the picnic?*
**V.7. Information that can rarely be indicated in any photo:** *Does the woman in the center have perfect pitch? What is the conversation about?*
**V.8. Questions with a false premise:** *What color is the horse? Which woman is punching the man? What colors are the boots?*
**V.9. Low-quality image:** As mentioned, these are common in VizWiz, though essentially non-existent in datasets such as VQA.
**V.10. Completely irrelevant:** *What is the capital of Alaska?*
**V.11. Not a question:** *Hello, this is another try*. These exist in some number in VizWiz.
**V.12. Nonsense (i.e., meaningless in any context):** What did the rug eat for lunch? Will the shadows set fire to the tree?
**V.13. Gibberish:** *What color are the margialigoelntest farbitlangefs?*

Some of the questions that I have put in categories V.1-V.4 and V.6 may well be in category V.5; that is, a sufficiently knowledgeable or perceptive viewer could answer them from the photo, though I cannot. There may well be people who can tell whether the woman in the center is wearing lipstick, who can identify the tree at the top just from the trunk, or who can judge accurately the distance from the tree to the pole, or who can tell whether the woman in brown is barefoot from the way she is sitting. But some of the questions are unquestionably impossible to answer; there is simply no way to tell whether the woman has perfect pitch or what the conversation is about (Of course, any question at all is answerable if you have the necessary knowledge about the individuals; if you recognize the woman in the center and you happen to know that she has perfect pitch, then you can answer the question, “Does the woman in the center have perfect pitch?”).

For categories V.1-V.7, the best answer would be “I don't think it's possible to tell” when that is the case, or “I can't judge” when I think that perhaps someone else could answer the question. In category V.8, it would be better to deny the premise—“There's no horse in the picture,” “No one is punching the man”—and better yet, when possible, to clear up the questioner's inferred confusion “Those aren't boots, they're black sandals.” As discussed above, the system developed by Mahendru et al. (2017) generates answers that deny the premise. For category V.9 the proper response is, “There is a problem with the picture.” For category V.10-V.13, the proper answer is “What are you talking about?” or “Are you OK?” AIs of general intelligence should likewise be able to give these kinds of answers.

## 2. Unanswerable Questions About Text

A number of recent papers, stemming from the seminal paper (Rajpurkar et al., 2018b), have studied the use of unanswerable questions about text as adversarial examples to probe the depth of understanding in QA systems. Rajpurkar, Jia, and Liang collected 53,775 unanswerable questions, using the following procedure: Crowdworkers were shown a 25-paragraph article drawn from SQuAD, an earlier database for text QA (Rajpurkar et al., 2018a). They were asked to create, for each paragraph in the article, between one and five questions that were about the topic of the paragraph and referenced some entity in the paragraph, but in fact were unanswerable from the paragraph (they were given an example as illustration). The questions thus collected were added to SQuAD creating a new dataset SQuAD 2.0 (originally called SQuADRUn).

Rajpurkar, Jia, and Liang tested a number of high-quality QA systems on SQuAD 2.0, and found that they performed significantly worse than on SQuAD; for instance, the F1 score on the best-performing system DocQA+ELMO dropped from 85.8% on SQuAD to 66.3% on SQuAD 2.0 (human performance was 89.5%).

QA datasets prior to SQuAD 2.0 had contained a fraction of questions that were unanswerable, due mostly to noise. These were mostly easy to detect as they did not have plausible answers. Jia and Liang (2017) had proposed a rule-based method for generating unanswerable questions; these were less diverse and less effective than the crowd-sourced questions created for SQuAD 2.0.

A number of papers since have taken up the challenge posed by SQuAD 2.0. The U-Net system (Sun et al., 2018) uses three components—an “answer pointers,” a “no-answer pointer,” and an “answer verifier”—and achieved an F-score of 75% on SQuAD 2.0. Hu et al. (2019) likewise used a multi-component system and achieved an F-score of 74.2%. Zhu et al. (2019) used a pair-to-sequence model to generate their own corpus of unanswerable questions, and by training a BERT-based model on these, achieved an F-score of 83% over SQuAD 2.0.

Rajpurkar et al. (2018b) also manually analyzed a small subcollection of the new unanswerable questions, and found that they fell into the categories shown in Table 1.

Tan et al. (2018) constructed a different dataset with unanswerable questions (SQUAD-T) as follows: They took questions from SQUAD and used Lucene to extract the seemingly most relevant text passage. If the text passage in fact included the answer to the question, it was included in the dataset as a positive example; if not, it was included as a negative example.

## 3. Comparing Categories of Unanswerable Questions

All the categories of textual unanswerable questions in Table 1, except T.7, are forms of “invalid premise,” and thus correspond to category V.8 of section 1.1; they categorize different strategies that can be used to construct a question with an invalid premise from text. One could certainly find, or construct, unanswerable visual questions corresponding to each of these. For instance, applying them to the images in top row of Figure 1, we could formulate such questions as:

V.8.1 **Negation:** Why isn't the ground wet?
V.8.2 **Antonym:** Why is the ground dry?
V.8.3 **Entity Swap:** What color is the shopping cart?
V.8.4 **Mutual Exclusion:** What is the pink electronic device?
V.8.5 **Impossible Condition:** What color shirt is the person facing us wearing?

In the absence of a text, however, these categories are much less distinct from one another.

The invalid premise questions constructed by Mahendru et al. (2017) include questions about objects not present in the image, such as “What color is the horse”? These are excluded from SQuAD 2.0 because their verbal analogue is too easy.

Going in the other direction: Categories V.1-V.5 and V.9 have to do with features of images not relevant to text. Categories V.9-V.12 have to do with low-quality data that is included in VizWiz but not in SQuAD or VQA.

Category V.7 “Information that can rarely be indicated in any photo” has no analog for text; if a question has an answer, then there is almost always some way of expressing that answer in language.

Category V.5, “Information that is indicated in the photo, to a viewer with appropriate general knowledge,” applies to text no less than to images. For instance, given the text of example T.4, “Union forces left Jacksonville and confronted a Confederate Army at the Battle of Olustee,” many of my American readers can answer the question “What war was the Battle of Olustee part of?” By contrast, fewer American readers, seeing the text “At the Battle of the White Mountain, the combined forces of Charles Bonaventure de Longueval and Johann Tserclaes defeated the army of Christian of Anhalt,” can immediately answer the question “What war was the Battle of White Mountain part of?” But, of course, there is no difference in kind here, only a difference in the general knowledge of a particular audience. In this case, the results might be quite different for a Czech audience.

I have not seen any discussion of this kind of question in either the QA or VQA literature. Presumably, the crowd workers rarely generate these, either as answerable questions or as unanswerable questions. AI researchers seeking to put together a reliable corpus for training and testing would naturally tend to avoid these; they can hardly be generated automatically in current technology, they are probably very hard to recognize, they probably have poor interannotator agreement among human judges. However, they are interesting test questions for systems that try to integrate question answering with background knowledge.

## 4. Other Related Work

Standardized tests, of various kinds, have included multiple-choice questions, or yes/no questions, asking whether a specific question can be answered from specified information. This is a regular part of the GMAT exam for business school, known as “data sufficiency” problems. Such questions were part of the math SATs at one date, but were eliminated because they were found to be too susceptible to coaching (Chipman, 2005)^2^. I don't know whether problems of this kind are included in any reading comprehension tests.

The earliest reference that I know to unanswerable questions in the AI literature is an example attributed^3^ to John McCarthy:

A. Is the President sitting down or standing up at this moment?

B. I haven't the faintest notion.

A. Think harder.

The point being that B *knows* that thinking harder won't help, but that it is not easy to say *how* he knows it.

Davis (1988, 1989) develops a logical theory, using a possible-worlds semantics in which the limitations of perception due to occlusion or limited acuity can be expressed and connected to a theory of knowledge. In principle, such theories could be part of a knowledge-based approach to identifying unanswerable VQA questions.

## 5. Unanswerable Questions in AI Generally

Any AI system can be viewed as a question-answering system for a narrow class of question. A machine translation system answers questions like “How do you express this English sentence in French?” A chess program answers the question “What is the best move in this state of the game?” Thus, some of the issues that arise with unanswerable questions in QA or VQA reflect more general issues with unanswerable questions for AI programs generally. What distinguishes QA systems is that the question is explicitly given in natural language, and that there is therefore a wide range of possible questions.

AI systems generally have a particular space of outputs considered useful, and they typically generate an output in this output space no matter what the input. The AI system may be entirely unsure that its answer is correct. It may have never seen a similar example. There may be no good output for the particular input; or the input may be meaningless; or the input may be random noise. Nonetheless, AI systems mostly will confidently output what they consider to be their best answer in this outputs space. When there is a problem with the input, the chosen output often has more to do with the AI's understanding of the output space than with the particular input.

In one widely reported incident a couple of years ago (Greg, 2019), someone discovered that if you asked Google Translate to translate, “dog dog dog dog dog dog dog dog dog dog dog dog dog dog dog dog dog dog dog dog” from Yoruba to English, the result was

Doomsday Clock is three minutes at twelve We are experiencing characters and a dramatic developments in the world, which indicate that we are increasingly approaching the end times and Jesus return.

(The omission of the period between “twelve” and “We” was in the output from Google Translate.) This particular example no longer works; Google Translate output now just echoes the input. But similar, if less entertaining, errors persist. For example, as of 1/17/2020, when asked to translate “margialigoelntest farbitlangefs” from English into Hebrew, Google Translate outputs “shodedei m'raglim rabim,” which means “many spy bandits.” If you ask Google Translate to translate it into Turkish, you get “margialigoelntest Instagram Hesabındaki Resim ve Videoları farbitlangefs” which means (I think) “margialigoelntest the Instagram account photos and videos farbitlangefs”^4^. As I will discuss below, in one form or another, this kind of behavior is very easy for AI systems to fall into, given the way that they are built.

**Figure 4:**
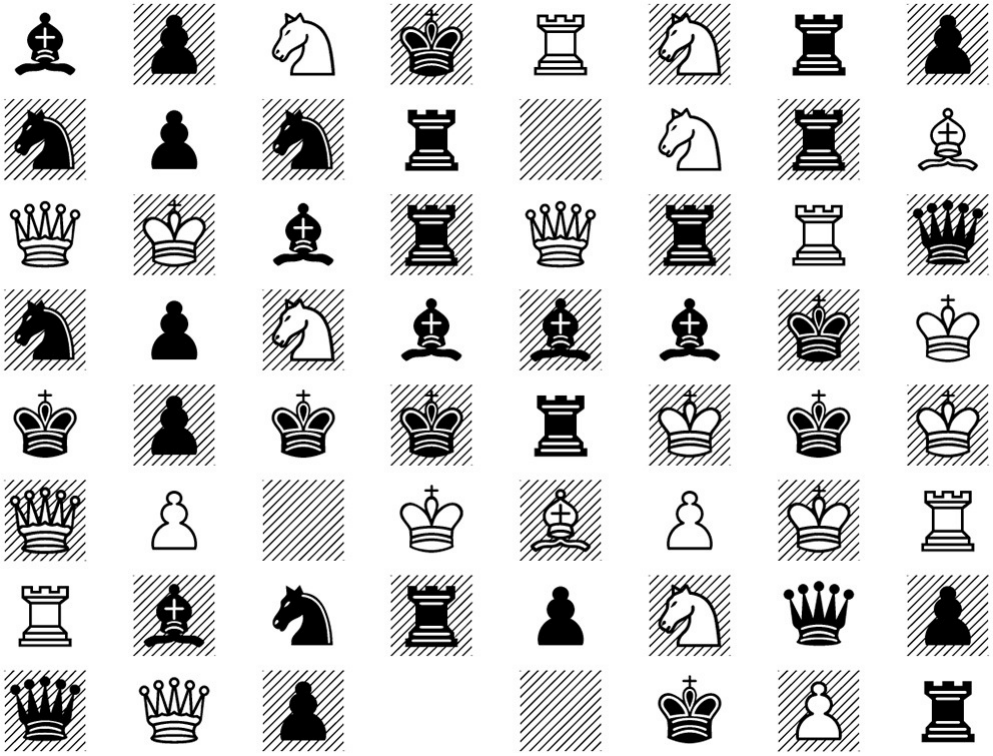
Unanswerable question in chess: What is the best move for white?

There are even unanswerable questions in chess. A chess program, such as the chess version of AlphaZero (Silver et al., 2017), will accept as input any of the 13^64^ arrangements of the twelve pieces and “empty” on the chess board as a legitimate input, and for any of these, it will output a move, because its output space is the class of moves. But the rules of chess are only defined for boards in which both players have exactly one king (If a player has two or more kings, do you have to put them all checkmate? Or to put one of them into checkmate? to take all but one and put the last into checkmate? The rules do not say, because the situation cannot arise). Therefore, in a situation like Figure 4, the proper response is “This problem is meaningless,” but AlphaZero will output a move^5^.

### 5.1. An Abstract Framework

It will be useful to setting up a general, abstract framework in which to discuss these issues.

Generally speaking AI systems, and for that matter computer programs of any kind for a particular task, the actual ultimate objective can be formulated as follows. There is a class *X* of inputs that are “reasonable” problems for *Q*. There is a class *Y* of possible outputs. The task defines a relation *Q*(*x, y*) meaning “*y* is a good output [or an acceptable output, or the best possible output] on the task for input *x*.” We assume that for every *x* ∈ *X* there is at least one *y* ∈ *Y* such that *Q*(*x, y*). Define *Y* to be the set of all good outputs for reasonable inputs: *Y* = {*y* | ∃_*x*∈*X*_ *Q*(*x, y*)}.

We now consider an ML-based AI system for this task. The construction of the system begins by the assembling a corpus *C*. Ideally *C* should be a representative sample of *X*, but often because of the way it is constructed, it is biased and omits large swaths of *X*. You randomly divide *C* into the training set *T* and the test set *S*. You then construct an architecture that takes arguments of form *I* and produces outputs of form *O*. *I* is often much larger than *C*; *O* can be equal to *Y*, or it can be much larger, or much smaller. You then apply the machine learning function to the training set *T* and learn a function Φ_*T*_(*x, y*) which is the system's judgment of how good *y* is as an output for *x*. Now, at inference time, given any *x* ∈ *I*, the machine will output argmax_*y*∈*O*_Φ_*T*_(*x, y*), the most suitable output (we ignore the difference between the ideal objective function and the answer that the mechanism actually outputs, which is not important for the analysis here). You test the machine over *S*; of course, this test can detect overfitting to *T* but not biases in *C*.

A few examples:

- In a speech recognition system, *X* is the set of all things that someone might plausibly say. *Y* is the set of meaningful natural language expression. *I* is the set of all waveforms. *C* is the corpus of speech samples that have been collected.
- In a chess-playing program *X* is the set of all game states that plausibly might come up in an actual game. *Y* = *O* is the set of all moves. *I* is the set of all placements of chess pieces on a chess board, including Figure 4. *T* is the set of games that the program has looked at in training.
- For an image categorization system based on ImageNet, *X* is the set of all reasonable images. *Y* is the set of all categories of objects that appear in images. *I* is the set of all pixel arrays. *O* is the set of ImageNet categories. In this case, *O* is considerably smaller than *Y*; that is, there are images of objects where a good answer is a category not in ImageNet.
- For high-quality VQA, *X* is the set of all pairs of a high-quality image together with an answerable question about the image. *I* is the set of all pairs of a pixel array and a character string. *Y* is the class of all reasonable answers to questions about images. *O* is the space of the possible outputs of the system—some subset of the space of all character strings.
- For textual QA, *X* is the set of pairs of a text together with an answerable question about the text. *Y* is the set of plausible answers to such questions. *I* is the set of pairs of character strings^6^. *O* is the space of character strings.

Ideally, a system should respond to an input *x* with either “It's impossible to tell” or “the question is meaningless” if the question is unanswerable; that is, if *x* ∈ *I* but *x* ∉ *X* (Our framework here is not rich enough to distinguish between these two answers). The system should respond, “I don't know” in either of the following cases:

- The system has no confidence in any possible answer: ϕ_*T*_(*x, y*) is small for all *y* ∈ *O* (this may be, of course, because the true answer isn't in the space *O* of the answers that the system can produce).
- The input *x* is so dissimilar to any part of the training set that the there is no reason for confidence that ϕ_*T*_ approximates *Q* at *x*. That is, you have no confidence that the learning method will do out-of-domain generalization with any quality. As an example, one can consider inputting a line drawing to a vision system that has been trained exclusively on photographs, or an audio of French speech to a voice-recognition system trained on English.

It is *possible*, of course, that the second case reduces to the first—that whenever the input is too far from the training set, that will be properly registered in the function ϕ_*T*_—but one certainly cannot assume that will hold in any particular case.

### 5.2. An Extreme Case

A case where *X* and *Y* vary to an extreme degree from *I* and *O* is the system for symbolic integration developed by Lample and Charton (2019) (henceforth LC) using seq2seq technology (Davis, 2019 is an extended critique). LC takes an elementary function *f* (i.e., a composition of the arithmetic functions, the trigonometric and exponential functions and their inverses) as input and is supposed to produce as output the indefinite integral of *f*, likewise expressed as a symbolic elementary functions. It was trained and tested over a corpus of elementary functions whose integral is also elementary and, over this corpus, achieved a higher success rate than state-of-the-art systems for symbolic mathematics such as Mathematica and Matlab.

The problem, though, is that, for the vast majority of elementary functions *f* of any significant complexity, the indefinite integral of *f* is non-elementary. For all of these, LC would happily return an elementary function as an output, and that answer would necessarily be wrong. And the class of “elementary functions whose integral is also an elementary function” is by no means a mathematically natural one.

So in terms of our framework: *X* here is the class of elementary functions. *I* = *O* is the class of combinations of elementary and arithmetic functions, both well-formed and ill-formed (the output of LC is occasionally an ill-formed expression and LC will accept an ill-formed expression as input without protest). *Y* is the class of elementary functions union the output “The integral of the input is non-elementary.” *C* is a corpus of elementary functions whose integral *is* elementary. LC achieved an almost perfect success rate over a test set drawn from *C*, but would almost never succeed over a test set drawn from complex expressions in *X*.

The unanswerability of a question like, “What is the integral of sin(sin(x))?” is in a different category from those we have discussed for QA and VQA. The question is meaningful—the function has an indefinite integral—but the answer is not expressible in the language of elementary functions.

## 6. Why Are Unanswerable Questions Important?

Why is it important to deal with unanswerable questions or invalid inputs at all? Why not just say, “Garbage in, garbage out” and leave it at that?

Sometimes, in fact, that's fine. There is no reason that the AlphaZero team at DeepMind should spend any time getting the program to respond correctly to the situation in Figure 4. They can be entirely confident that the only way their system will ever be used will be in the context of an actual chess game, and equally sure that this situation may never arise in an actual chess game.

But often it is important to respond properly to unanswerable questions and invalid inputs, for a number of reasons. First, once a program is deployed to the real world, invalid inputs do often occur, and they have to be flagged. Serious conventional programs for general use are always built to address the problem of invalid inputs; it's a basic principle of software engineering. If a program bombs out without explanation when it gets invalid input, that's not great, but it is much worse if the program returns a plausible-looking answer, because then the user has no warning that there is a problem. A compiler which, analogously to LC, produced correct executable code when the source was syntactically correct, but also produced random executable code when the source is syntactically incorrect would be a disaster.

The same applies for AI applications. It's a ridiculously far-fetched example, of course, but suppose that a public figure were to tweet something like, “Despite the constant negative press covfefe,” and you were tasked with translating that into a foreign language. There are various options you might consider, and it's not clear what would be best, but simply rationalizing it to a phrase that makes some sense would not be a good option.

Similar considerations apply to answering unanswerable questions in QA and VQA. As we have seen, invalid inputs, both in image and in question, are fairly common in VizWiz. More generally, suppose that you had a powerful VQA system that could reliably answer legitimate questions, but also answered unanswerable question. Now, someone maliciously or not, gives the system a photo of a tall, thin African-American man shaking hands with some terrorist *X*, and asks “What is Obama doing with *X*”? The AI system answers “Obama is shaking hands with *X*.” The mis- or disinformation now gets posted on social media, with the imprimatur of the AI system.

Second, when AI outputs are being used as guides to action, then it becomes extremely important to know how reliable they are. You want to avoid deciding on an expensive or risky action on the base of unreliable information. This is all the more important, because it is often completely impossible to anticipate how an output will be used. A passage is mistranslated, a person in an image is misidentified, speech is mistranscribed; OCR misreads a text; the mistaken information is uploaded to Web, or posted on social media; and then there is no telling what anyone will do with it.

Third, unanswerable questions can be used as adversarial examples that give a means of probing the depths of an AI system's understanding. If a VQA system confidently answers “brown” to the question “What color in the horse in Figure 3?” or states that the woman in the brown shirt is barefoot, then that raises eyebrows. This kind of probing is the primary justification for including unanswerable questions put forward in most of the current literature in VQA and QA.

This is probably useful, but a couple of cautions should be noted, in both directions. On the one hand, if a system has been trained to respond correctly to unanswerable questions, that may indeed reflect a deeper understanding, or it may just mean that the system has learned “Clever Hans” tricks for that category as well. Conversely, even if a system answers “brown” to the question, “What color is the horse?” or “Yes” to the question, “Is the woman in the brown shirt barefoot?”, that may not reflect any gap in the system's understanding of horses, brown, or bare feet. The gap may simply be in its understanding of what is best to do when confronted with an unanswerable question (school children likewise often guess randomly when confronted with this kind of “trick” question, if they are not expecting it).

Fourth, under some circumstances, including unanswerable questions in training may improve performance even on other tasks. Mahendru et al. (2017) report that including unanswerable questions in their training set had the effect of improving performance on tasks involving compositional reasoning.

Fifth, it is important to keep unanswerable questions as a category in mind when comparing human beings or conventional computer systems against AI systems. Human beings generally deal with unanswerable questions flexibly and effectively; conventional software is often written to give useful output for invalid inputs; AI systems mostly avoid the issue altogether. The claim that “Chess programs are always better at analyzing a chess board than humans” is inarguably mostly true but comes with a very small asterisk for the reasons discussed above; the claim that “Watson can answer Jeopardy-style questions better than human competitors” comes with a significant asterisk; the claim that “Lample and Charton's symbolic integrator outperforms Mathematica at symbolic integration” comes with a very large asterisk, and indeed is more false than true (Davis, 2019).

Finally, identifying what information is missing and why it missing is the first step toward taking action to obtain the information. If the image in Figure 3 were the current perception of a robot, who for some reason needs the answers to the question, there are a number of actions it might take. It could move its camera, or move to a new viewing position, or move some occluding barrier, or wait until the picnickers stand up, or ask politely. Which of these would work and would be appropriate of course depends on the particular question and circumstance, and choosing the answer depends on a meta-level understanding of what kind of information can be gotten under what circumstances.

The ability to recognize input as ill-formed or nonsensical is one aspect of understanding what the input means when it is well-formed and meaningful. The ability to realize that a picture or text does not provide a particular kind of information is part of understanding what kind of information it does provide. The ability to realize that you do not have the experience, knowledge, information, or cognitive capacity to be sure of any answer is an aspect of self-knowledge critical in reasoning, acting, and learning.

## Data Availability Statement

The original contributions presented in the study are included in the article/supplementary materials, further inquiries can be directed to the corresponding author.

## Author Contribution

The author confirms being the sole contributor of this work and has approved it for publication.

## Conflict of Interest

The author declares that the research was conducted in the absence of any commercial or financial relationships that could be construed as a potential conflict of interest.

## Footnotes

^1^Regrettably, the acronym VQA refers both to the general area of research and to this specific, widely used, dataset.

^2^Interestingly, there were studies that suggested that female students did better than male students on these questions.

^3^I mentioned this myself in Davis (1990), but I did not record there, and now do not remember, where I got it from—perhaps word of mouth—and I cannot find any other mention of it Googling.

^4^In fairness, Hebrew and Turkish are somewhat exceptional in this regards. In most languages Google Translate simply echoes the input, even in languages that don't use Roman characters, like Arabic and Russian; it does not attempt to transliterate.

^5^It may be noted that only a tiny fraction of such arrangements of chess pieces have one king for each player. Thus, for the vast majority of chess boards—though, of course, not of chess boards that ever occur in practice—a human being gives a better answer than the best chess-playing programs.

^6^In many recent systems, there is a preliminary step of converting words into word embeddings; so, arguably, *I* would be the set of pairs of sequences of word embeddings.
